# The Cognitive–Affective Social Processing and Emotion Regulation (CASPER) model

**DOI:** 10.1038/s41386-025-02224-x

**Published:** 2025-09-04

**Authors:** M. Catalina Camacho, Elina Deshpande, Michael T. Perino

**Affiliations:** Department of Psychiatry, Washington University School of Medicine, St. Louis, MO 63110 USA

**Keywords:** Social neuroscience, Predictive markers, Cognitive neuroscience, Emotion

## Abstract

Humans are intensely social creatures. It is therefore not surprising that many psychiatric disorder criteria include social dysfunctions; however, overlapping presentations and diverse, complex etiologies make treating social dysfunctions difficult. Here, we introduce the Cognitive Affective Social Processing and Emotion Regulation (CASPER) model. CASPER integrates research from social psychology, cognitive neuroscience, and developmental science to describe how real-world social processing unfolds and is associated with differing psychiatric social cognitive neurophenotypes. Briefly, social processing can be broken down into the following cognitive steps: identifying relevant social cues, attending to related cues, interpreting cues, and adjusting behavior appropriately. Each of these steps is influenced by the individual’s affect and goals in the moment, which in turn influence which social concept or schema is activated for that interaction. Concepts are formed across development as we learn social skills and gain life experience. This model therefore links early experiences to social dysfunction “in the moment”. The goal of this model is to provide a testable scientific framework for psychiatric research into social dysfunctions, as well as provide a model that generates new treatment targets for improving interventions and reinterpreting differences based on the extant research.

## Introduction

As humans, we are highly social creatures whose well-being is tightly intertwined with our ability to successfully navigate our interpersonal relationships. Indeed, many psychiatric disorders have social impairments included in their DSM symptoms such as anxiety, mood, and conduct disorders, while other disorders have symptoms which may not be inherently social but may cause challenges in social functioning, such as posttraumatic stress disorder, schizophrenia, attention deficit hyperactivity disorder and obsessive-compulsive disorder. Many of our most effective cognitive and behavioral treatments already target social cognitive processes, such as parent-child Interaction therapy, cognitive behavioral therapy, or the use of token economies to treat school misconduct. Furthermore, social skills and cognition develop over a protracted period in childhood, concurrent to neurodevelopment of correlated brain systems as well as the windows when many social dysfunctions emerge. Yet, neurodevelopmental context is rarely included in clinical evaluations. Characterizing the transdiagnostic and unique neurobiological basis of social dysfunction in psychiatric disorders—expanding on the research domain criteria approach [1] and using a neurodevelopmental lens—could therefore provide novel, key insights for refining our existing understanding and developing novel interventions [2, 3].

Real world social cognition is a dynamic and complex process involving first identifying, and then selectively attending to, relevant external stimuli, interpreting these cues, and adjusting behavior appropriately. These cognitive processes occur in the context of individuals affective states and goals, which are in turn highly dependent on both recent and long-term experiences. Social scientists have described models to fit many of these processes together, such as the Social Information Processing model [4, 5], the Process Model of Emotion Regulation [6], and attachment theory [7]. These empirically supported psychological theories have provided clear examples of how experiential learning impacts the social cues we perceive, seek out, and interpret, with clear links to future behaviors. Linking the development of these psychological processes to neurobiological development is still in its nascency, however, and these social-cognitive models do not engage with important neurodevelopmental context. For instance, in attachment theory, experiential learning between infant and caregivers is thought to promote models of social behaviors and expectations and, implicitly, how these impact the brain. However, explanation of how these behaviors are substantiated in the brain and impact later neurodevelopment are unclear. In theory, based on decades of cognitive neuroscience work, the cognitive processes engaged during social interactions requires the engagement of many functional brain networks, including sensory, attentional, and cognitive control networks. For the past 15 years or so, the neural basis of basic social cognition has been primarily linked with the default mode network (DMN) [8–11]. The DMN has been shown to activate during mind-wandering, autobiographical memory recall, and when making inferences about another person’s intentions [12], as well as during linguistic semantic processing [13]. However, while these functions are undoubtedly carried out during social interactions, they do not encompass the full social information processing model. Indeed, recent naturalistic fMRI work—which presents social information with full context—has found diverse, brain-wide activation patterns to specific emotion concepts [14], affective states [15, 16], and processing goals [17] spanning many networks beyond the DMN. Context, goal, and affect-specific activation across networks is consistent with our theoretical understanding of the social information processing model, with many diverse cognitive functions needed to navigate our social worlds. A model that integrates how neural models are influenced by experience *and* is integrated with social science models of human interaction and cognition is needed to capture such complexity.

The goal of the Cognitive–Affective Social Processing and Emotion Regulation (CASPER) model is to provide a clear framework linking social psychology, cognitive neuroscience, and neurodevelopment to better understand the neurobiological underpinnings of social dysfunction in psychopathology. We posit that diverse neurophenotypes likely arise from differences in one or more components of the model, which then impacts downstream processes (e.g., differences in attention influencing regulation capabilities). Understanding how differences in these processes link to psychiatric phenotypes will pay dividends for improving our treatment of psychopathology. To support these assertions, here we first describe the CASPER model in depth. Next, we provide two exemplars of how the CASPER model can be used to characterize the neurodevelopmental basis of psychiatric social dysfunction as well as the extant evidence in support of this formulation. Finally, we describe the broad clinical utility of the model for guiding research and eventual treatment for social dysfunction in psychiatric disorders and future directions. It is important to note that we conceptualize the brain in this model largely in terms of cognitive networks to provide a common heuristic for linking neuroscience and cognitive psychology research. We therefore interpreted prior research largely using this framing (when appropriate), even if the research was conducted using a regions of interest or brain mapping approach. We hope this framework will provide a common system for investigating and treating social impairments in psychiatric disorders within the neurodevelopmental context that these behaviors manifested under, recognizing that many aspects of dysfunction likely reflect short-term neural adaptations to specific experiences. It is our goal that in providing this framework, researchers can systematically test this model to elucidate the neurodevelopmental underpinnings of social dysfunction.

## Defining the CASPER model

### Introducing CASPER

**Fig. 1:**
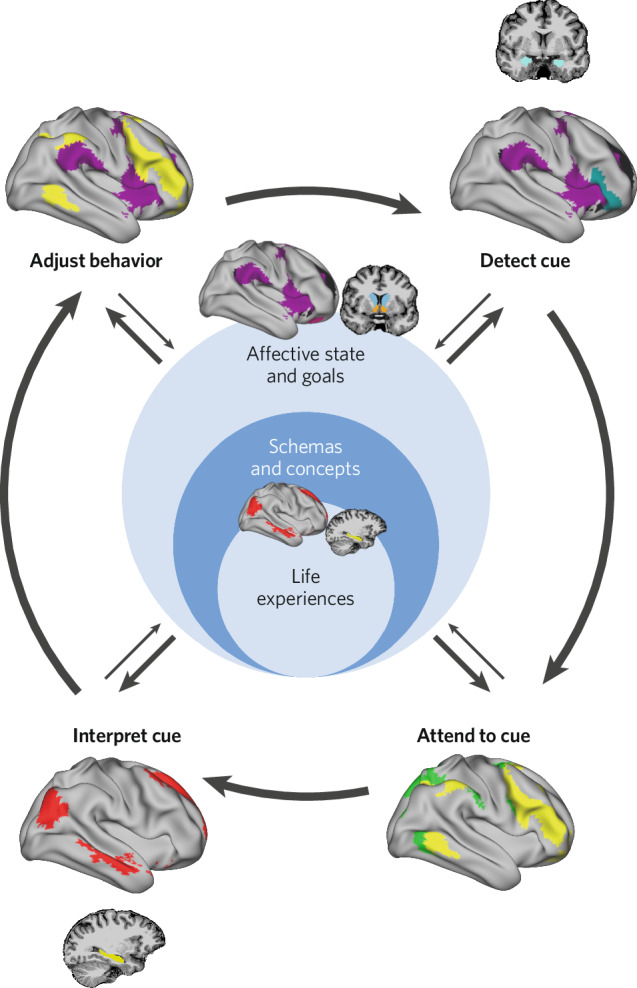
The cognitive affective social processing and emotion regulation (CASPER) Model. This model combines social psychology, cognitive neuroscience, and neurodevelopment to inform a model of how social cognition unfolds in the moment.

The CASPER model (described in brief in Fig. 1) describes the cognitive processes that our brains minimally undergo to navigate our social worlds as well as the brain systems theorized to support each process. The cognitive processes that are engaged are highly influenced by a combination of prior experience shaping expectations and response patterns (i.e., neurodevelopment) and current affective states or goals. In mature organisms, the brain is primarily functioning as a predictive machine, anticipating upcoming cues and preparing interpretations and responses. For instance, we have expectations for how certain social interactions (such as ordering coffee) unfolds. The way our brains predict how interactions unfold is most obvious when these expectations are violated (e.g., if the barista were to scream in response to the coffee order). Processing that specific interaction is still a process of detecting, interpreting, and responding to external cues, but these range of these cognitive processes are shaped by expectation for maximal efficiency in parsing our complex social worlds. It is unclear how these predictive models of our social worlds are formed across development [18, 19], however and the mechanisms that result in maladaptive social behaviors are unclear. In this section, we break down each cognitive process, including evidence for individual differences in these processes that statistically explain variation in psychiatric functioning (see Table 1), and describe how neurodevelopmental research can elucidate the mechanisms underlying social dysfunction.

**Table 1:** Evidence for dysfunctions in CASPER processes across psychiatric disorders.

Disorder	Evidence Category	Experiences/Concepts (Hippocampus, MTL, DMN)	Affect/Motivation (Striatum, VMPFC, OFC, Insula)	Detecting Cues (Amygdala, VAN, SAL, CON)	Attend To Cues (FPN, DAN)	Interpret Cues (Hippocampus, MTL, DMN)	Adjust Behavior (FPN, CON)
Depression	Cognitive	Childhood maltreatment confers ↑ risk of later diagnosis; ↓ emotion differentiation [41, 189]	Childhood irritability present ↑ risk of later diagnosis [269]	↑ attentional bias to negative stimuli [269]	↓ selective attention [270]	↑ interpretation of ambiguous stimuli as negative [89, 271]	↓ flexibility in cognitive control [122]
	Neural	↑ resting DMN FC; ↓ hippocampus volume [163, 185]	↓ striatum activation to rewards [272]	Mixed associations with resting VAN FC [163]	Mixed associations with resting DAN FC [163]	↑ resting DMN FC [163]	
Anxiety	Cognitive	↓ emotion differentiation [189]	Avoidance of changing mood states [69]		↑ attention to threat [273]	↑ interpretation of positive/neutral stimuli as negative [122]	↓ flexibility in interpretation, regardless of disconfirming information [122]
	Neural			↑ activation in vIPFC to longer-duration threats [273]			
Bipolar Disorder	Cognitive	↓ emotion labeling accuracy in high-risk youth [274]	↑ approach motivation [275]		↓ selective attention [276]		
	Neural		↑ striatum activation to reward [272]	↑ amygdala & VAN activation during emotion face processing [165]		↑ DMN activation during emotion face processing [165]	
Conduct Disorder	Cognitive	Adolescent diagnoses predict ↑ adult psychiatric impairment; Early adversity ↑ risk for aggression [208]				↓ affective morality [277]	↑ in IE and CU tendencies; ↑ in impulsivity, violent and damaging acts [205]
	Neural	↓ DMN connectivity in response to fixed stimuli [255]	↓ structural connectivity between Amygdala-OFC [256]		↓ FPN-DMN correlation in response to empathy [248]		
Psychopathy	Cognitive				↓ attentional shift to salient cues (functional neural)	↓ salience during morally aversive scenarios (neural networks)	↑ desire to return to homeostasis through aggression [261]
	Neural	↑ activation in DMN during self-referential processing [246]		↓ in visual processing and somatosensory networks [243]	↑ FPN activity during externally focused tasks [246]	↓ fear processing in MCC [278]	
Borderline Personality Disorder	Cognitive	Previous violence confers ↑ risk of aggression [279]			Impairment of attention orientation [280]	↑ attentional control in free-viewing [281]	↓ focus on positive stimuli; ↑ in suboptimal decision making; ↑ sterile response leads to automatic interpretation of cues as negative [281]
	Neural	Mixed associations with DMN activity; ↓ in sub-cortical to cortical FC [282]	↓ VMPFC activity to negative emotions during inhibition [280]	↑ activation in VAN across sensitivity analyses; ↓ activity in visual areas leading to reduced emotion accuracy [283]			
Intermittent Explosive Disorder	Cognitive		↑ desire for anger; ↓ desire for compassion [284]				
	Neural						

Evidence is organized into either cognitive (e.g., from behavioral studies) or neural (e.g., from neuroimaging studies) to faciliate integrating evidence across disciplines for each component of the model and each syndrome.

For this table, review papers and meta-analyses were prioritized for interpretation. Empirical work is not exhaustively listed, though there is extensive additional evidence. MTL medial temporal lobe, DMN default mode network, VMPFC ventromedial prefrontal cortex, OFC orbitofrontal cortex, VAN ventral attention network; SAL salience network, CON cingulo-opercular network, FPN frontoparietal network, DAN dorsal attention network, FC functional connectivity.

### Experience and concepts

There has been extensive cognitive research suggesting that our brains rely on prior concepts to shape our expectations and therefore behaviors in social situations [20–22]. A social concept is a model of how interactions unfold which shapes expectations, such as our shared concept for ordering coffee at a shop or for catching a flight through a commercial airline. Even young infants demonstrate an expectation of others’ behavior that links emotion cues to specific social situations [23, 24]. These concepts are shaped by prior experiences, evidenced by regional and cultural differences in both emotional expression and behavioral responses to common social situations [25, 26]. Both human and animal work suggests that the hippocampus plays a key role in encoding experiences in cortex—likely through back propagation [27, 28]—for future access [29], and emerging work in humans suggest a hippocampus–medial temporal lobe–default mode network (DMN) circuit is implicated in accessing social concepts [30, 31]. This recent human work mirrors extensive animal work that demonstrates schema representation in the hippocampus and DMN [32–34]. While mechanistically there is the greatest evidence for the hippocampus–DMN circuit to be implicated in learning concepts, it should be noted that there is evidence for activation to common concepts to be present in diverse brain systems in mature organisms, such as visual cortex for visually presented concepts [14, 35]. This suggests that the hippocampus and DMN play a key role in encoding experiences into long-term concepts, which over time can alter how other cognitive systems function in specific social situations.

There is extensive evidence that early experiences shape hippocampal–DMN circuitry [36, 37] and that alterations in these circuits [38, 39] as well as adverse early experiences [40–43] are associated with later psychopathology. Smaller hippocampi and greater hippocampus–DMN connectivity have been found in adolescents and adults who have experienced early life adversity [38, 39] and recent work has demonstrated that unpredictable early environments are associated with alterations in medial temporal lobe regions and increased likelihood of developing later psychopathology [37, 44]. Insults within these circuits impact social concept learning through statistical learning. Early and middle childhood are critical periods for foundational social development—including developing basic concepts of functionally distinct emotions [45, 46], meaning adverse child exposures may have outsized impact on still-developing social concept formation. For instance, adverse experiences can produce short-term adaptations in these brain systems that are beneficial to the child in the near term (e.g., interpreting ambiguous social cues as potential threats), but confers greater social dysfunction and psychiatric risk in the long-term (e.g., negative interpretation bias). Together, this suggests a strong need for taking a neurodevelopmental lens to better understand how early experiences shape social concepts in children who develop psychopathology.

### Affect and goals

The way we feel in conjunction with our current goals shape the cognitive lens we use to process information during social interactions. Experimental research has shown that both affect and motivation can shape interpretation of ambiguous information [47–49] and influence semantic processing [50] and decision-making [51]. Practically, affect and motivation are difficult to disentangle empirically, since they are highly inter-related cognitive processes such that manipulating one often induces changes in the other. There is extensive evidence that motivational and affective brain systems are also highly intertwined, with evidence for each being encoded in or originating from the ventromedial prefrontal cortex, orbitofrontal cortex (OFC), anterior cingulate, anterior insula, and the ventral striatum [9, 52–55]. These regions are part of the cingulo-opercular (CON) and salience (SAL) networks [56–58] and are connected to midbrain, brainstem, and peripheral nervous system structures that modulate arousal and vigilance, such as the hypothalamus [59, 60]. Broadly, pleasure is encoded in the OFC and ventral striatum [61, 62] while unpleasant affect is encoded more so in the insula [63, 64]. All kinds of affective states are associated with activation in the VMPFC and anterior cingulate [15, 61]. Motivation-related cognition has been shown to be encoded in the anterior cingulate and OFC [65]. Because these systems are so interconnected and perform highly dependent cognitive functions, it is reasonable to think that an imbalance in either system can influence the other, altering how downstream higher order cognitive functions are performed. For this reason, we position “affect and goals” as a mediator for which social concepts or schemas are accessed and used in social cognition in a given moment. Importantly, given that this mediator is like strongly impacted by experience, it can be understood as a reflection of, and adaptation to, lived experiences rather than an explicit measure of dysfunction.

Persistent mood states and alterations in reward sensitivity or motivation are hallmark features of several disorders such as depression, bipolar, anxiety, and schizophrenia [66]. Specifically, adolescents and adults with depression have been shown to have reduced sensitivity to social reward marked by deriving less pleasure from positive social interactions and reporting less motivation to seek out positive social interactions [67, 68]. Anxiety disorders in general—but particularly generalized anxiety—are also associated with reticence to change one’s affective state, resulting in sustained negative mood states [69, 70]. Motivation and goals can alter how an individual is engaging in social interactions. For example, aggressive individuals often report that gaining power (relative advantage over others) motivates their behavior [71], and that they primarily value having a dominant social position [72]. Related phenomena (e.g. psychopathic traits) have largely shown low affiliative drive [73] and a preference for valuing dominance above other social values [74]. Interestingly, some neuroimaging work has found that such traits relate to evoked activity in reward regions in the striatum [75–77] while viewing social aggression, suggesting baseline differences in how affect and goals are acted upon may play an outsized role in aggressive behaviors.

### Cue detection

Our brains are bombarded with a milieu of sensory information that we must instantly make sense of and act on. A very early cognitive step in social processing therefore involves identifying which sensory cues are relevant to process consciously and attend to. Sometimes referred to as salience processing or salience detection, cue detection involves stimulus-driven brain systems that are heavily interconnected with systems of arousal and affect. Specifically, the anterior insula, amygdala complex, VMPFC, anterior cingulate, and ventrolateral frontal cortex [78–80] have been implicated in salience processing and have been referred to as the ventral attention (VAN), SAL, or CON networks depending on the reference atlas [81–83]. Salience gating can have critical downstream consequences for social processing, evidenced by lesion and optogenetic studies of these regions. For instance, there is evidence that the amygdala has a key role in detecting socially threatening information (though is not critical for social cue detection in general—see [84, 85]) such that lesions of the amygdala are associated with altered attentional prioritization of threatening cues [86] and less engagement of responsive prosocial behaviors [87]. Alterations of these systems therefore “gate” the information that enters in our conscious awareness, which in turn influences our interpretation of the situation and what we think is the appropriate response.

Alterations in salience and stimulus-driven processing have been found in individuals with anxiety disorders [88] and to some extent in individuals with depression [89, 90], though it is unclear how much this is explained by the high co-occurrence of anxiety with depression (see for example [91–93]). Specifically, individuals with anxiety disorders attend faster to threatening or ambiguous social stimuli and tend to take longer to disengage from these stimuli [94, 95]. This suggests hyperfunctioning of these salience and stimulus-driven attentional systems during social processing. There is emerging evidence for these neural alterations: anxiety symptoms have been associated with hyperconnectivity of the SAL, CON, and VAN [96] and increased activation of these networks during attentional processing [97, 98], suggesting increased involvement of these networks in cognitive processes for individuals with anxiety. Recent work using movie-watching to capture more naturalistic social processing has supported this theory [99, 100]. Reduced focus on distress cues in others—as evidenced by a reduced emotional “blink” [101]—has also been a consistent finding in aggressive youth [102], along with reduced activation in CON and SAL regions [103], suggesting these alterations may be reflected across various diagnoses with differing phenotypic expression. Downstream differences in social processing may ultimately be attributable to alterations in this early phase of cognitive processing.

### Attending to cues

Once relevant cues are detected in our environments, we must engage in goal-directed attentional processing to identify additional cues that provide context to the initially detected cue. For example, if a parent notices a brief shift from a smile to a frown on their child’s face, they may take a closer look at how their child is moving to infer discomfort or pay closer attention to what kind of conversation or play behaviors align temporally with their child’s changes in affect. Engaging these top–down, goal-directed systems involves suppressing attention to other cues that may otherwise be salient (e.g., another family member entering the room). Research with eye-tracking attentional tasks has shown that the dorsal attention (DAN) and frontoparietal network (FPN) are highly engaged with goal-directed attentional processing [104–106]. The DAN is canonically composed of the frontal eye fields, intraparietal sulcus, and lateral occipital cortex, all of which are regions of higher order visual or motor processing [104, 106]. The FPN typically includes regions of the dorsolateral prefrontal cortex, intraparietal sulcus, and middle temporal gyrus and has been associated with cognitive flexibility [107–109], a cognitive process that is often engaged during naturalistic goal-directed attention. The interplay between goal-directed attention during social processing, goals, and stimulus-driven attention can dramatically alter how a situation is ultimately interpreted and how the individual chooses to respond. Further, these systems are likely highly influenced by predictions of how the social situation is expected to unfold. This is evident in when we miss otherwise salient stimuli, such as missing that an acquaintance walked past you are walking down the street with a date versus walking by yourself.

Differences in goal-directed attention are theorized to alter disengagement from or suppression of stimulus-driven attention processes in line with the person’s goals, which can result in several downstream consequences. Differences in goal-directed attentional processes in individuals with psychiatric symptoms could be classified as alterations—reflecting dysfunction in the balance or engagement of attentional systems—or simply differences reflecting differences in goals. For example, the hyperactivation of stimulus-driven attention in individuals with anxiety described in the previous section could be a result of hypoactive goal-directed attention systems [88]. This theory is supported by previous work showing that higher attentional control in infants is associated with less anxiety-related behaviors later in childhood [110, 111] and that improving goal-directed attention is associated with decreasing anxiety symptoms [98]. For some social dysfunctions, differences in during goal-directed attentional processing may be better attributed to differences in the goals themselves. For example, psychopathy is a personality syndrome that is characterized by a tendency to exploit others rather than to protect, empathize, or otherwise engage in prosocial behaviors [112]. As such, failure to focus attention towards such distress cues may just as easily reflect misalignment with goals as it does with an inability to do so [113]. Researchers can tease apart differences in basic functioning of attentional systems versus differences in attention because of differences in goals by measuring attention in clinical and comparison samples across contexts, such as during “cold” attentional processing, attention during a specific social reward, or attention when interacting with a person expressing vulnerability.

### Interpreting cues

Once relevant cues are identified, the next cognitive step is interpreting these clues in the context of goals and prior experience in that given situation or with those persons. Interpreting social cues—especially those that are ambiguous or subtle—is an ability that is refined across development, with children learning to link cause and effect to social cues starting in infancy [45, 114]. This involves memory, relational, and semantic processing which are primarily carried out in the medial temporal lobe, hippocampus, and the DMN [115, 116]. Specifically, current social concepts or schemas relevant to the situation as well as specific prior experiences are rapidly accessed and used to interpret the intentions and actions of others. The interpretation step of social processing is the most intertwined with the rest of the cognitive steps across multiple timescales in the CASPER model as a result, and in turn is likely the most malleable across the lifespan as individuals gain increasing social experience.

Alterations in the interpretation phase of social processing may stem from several factors. For example, extensive research has demonstrated that anxiety and depression are associated with negative interpretation biases of ambiguous information across information types [89, 117–120]. This may be due to altered mood states influencing cue salience—such as weighing the lack of smile cues over the lack of frown cues when looking at a person quietly listening. Alternatively, this bias could be due to negative life experiences, which have reinforced that relatively neutral expressions in others are likely masking negative intentions rather than denoting a low arousal state in the other person. Finally, a third possible explanation for interpretation biases is alterations in upstream processing during social interactions. For example, one risk factor for social anxiety is childhood behavioral inhibition [121]. Individuals with social anxiety are more likely to have a negative interpretation bias [122], which is thought to be one downstream developmental result of negative attentional bias observed in inhibited children [123–125]. Inhibited children scan static pictures of emotional faces similarly to their peers, however they look to strangers’ neutral expressions fewer times and for longer fixations when interacting with a real person [126, 127]. Context-specific differences in social attention may therefore partially explain differences in interpreting others’ social cues.

### Adjusting behavior

The final cognitive step in social processing is adjusting behavior to better align with one’s goals and in response to others’ cues. This can involve adjustments in movement, tone, or emotion regulation, all of which involves exercising cognitive control. While the specific execution of cognitive control involves coordinating functions of other networks such as the expressive language network to adjust speech or the motor network to move, the core function of cognitive control has been shown to largely fall under the CON, and FPN [107, 116, 128]. Prior work examining the neural basis of cognitive control suggests a close interplay between these networks, where the CON maintains a course of action towards a goal while the FPN underlies cognitive flexibility and task-switching [108]. There is evidence that these systems are engaged during social processing as well, particularly during situations that require effortful control such as during emotion regulation [129–131]. The specific way that adjusting behavior is carried out varies by context and with developmental stage, as is discussed more in the next section.

Deficits in cognitive control is a feature of many psychiatric disorders, likely closely linked to impairment broadly [132] and difficulty regulating emotions specifically. One example of a psychiatric disorder marked by differences in cognitive control is schizophrenia. Specifically, schizophrenia is marked by differences in cognitive control across varied contexts, including when engaging working memory and adapting to changing goals or rules [133]. It has been posited that these cognitive control deficits interact with emotion processes, resulting in social cognition alterations observed in individuals with schizophrenia [134]. A transdiagnostic symptom also related to issues with self-regulation is irritability or aggression, which is often defined as an outsized negative emotional response to a blocked goal, though the specific definition can vary for each disorder [135]. Similar to anxiety, there is evidence that interventions targeting top–down cognitive control processes can reduce irritability symptoms in youth [136], suggesting that irritability may be explained in part by decreased engagement of cognitive control systems during social processing.

### Building CASPER across development

**Fig. 2:**
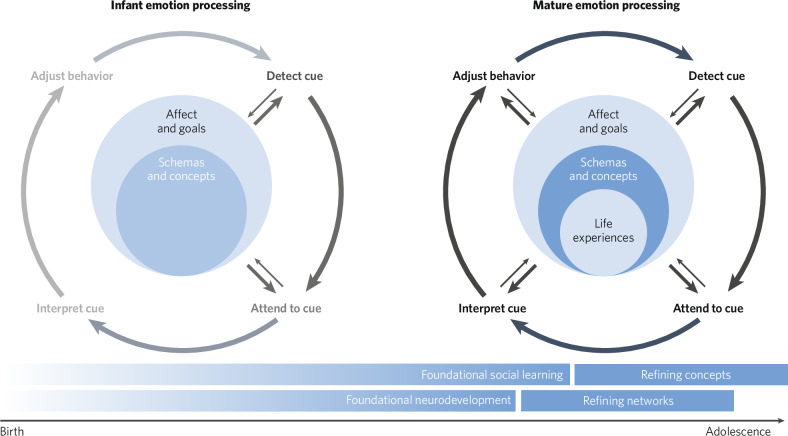
The developmental course of the CASPER model. At birth, humans are highly stimulus driven, corresponding to relatively earlier development of sensory and salience systems. As children develop, the brain forms a predictive model of social interactions where prior knowledge and experience shapes how we function socially.

Applying neurodevelopmental approaches (Fig. 2) to the study of psychiatric disorders could elucidate which CASPER process most directly influenced the development of specific social dysfunctions, providing novel targets for intervention. The order in which CASPER cognitive processes are engaged during social processing is also the rough order in which these processes develop. Sensory systems develop the most rapidly, for example, followed by higher order sensory/language and salience systems, while associative networks—which include those that underlie higher-order cognitive processes involved in the attention, interpretation, and behavior adjustment phases of the model—take the longest to fully mature [137, 138]. How these brain systems and cognitive processes develop is highly interlinked, such that early alterations of one system can influence the development of another. Given the highly protracted development of social processing, this means that there are countless opportunities for individual differences to emerge. Automatic self-regulation (as opposed to effortful emotion regulation), for example, is a function associated with the DMN [8] and is not engaged near birth, evidenced by low intensity negative stimuli inducing distress in infants but not in older children, adolescents, and adults [139]. In preschoolers and younger school-aged children, FPN activation is observed while processing similar negative stimuli presented in movies [140, 141], which is lessened in older school age children [14] and not observed in adults [35]. Across development, DMN functional connectivity increases [142–145] while the role of the DMN in negative emotion processing changes [14], likely underlying refinement of negative emotion cue concepts and increasingly mature understanding of social cues [146–148]. Taken together, this suggests a close interplay between the FPN and DMN in the development of automatic self-regulation in the face of lower grade negative emotion cues. Considering how common alterations of the DMN and FPN are in psychiatric disorders [149, 150], it is possible that alterations in FPN and DMN developmental trajectories is a mechanism through which early experiences confer risk for emotion dysregulation. CASPER can be used to test this hypothesis by characterizing how each specific process is unfolding across development and influencing downstream functioning. Specifically, tailoring longitudinal designs to assess how stressors during sensitive periods lead to within-person functional network changes will be key to empirically testing hypotheses generated through the CASPER framework.

We propose that taking a neurodevelopmental lens is particularly critical for understanding social dysfunction causing significant distress. The need to take a neurodevelopmental lens to understanding psychopathology in general has been extensively reviewed elsewhere (for example [151, 152]), and many of these arguments can be applied to understanding social dysfunctions specifically. For instance, disorders that are closely linked to alterations in earlier-developing social processes, such as anxiety which is most associated with alterations in the “cue detection” step, also tend to have earlier ages of symptom onset as compared to disorders linked to differences in downstream social processing [153, 154]. It is also perhaps not surprising then that there is evidence that earlier ages of onset of psychopathology is associated with worse outcomes [155]. In the next two sections, we illustrate the utility of the CASPER model—including the neurodevelopmental basis of the model—in parsing heterogeneity in each internalizing and externalizing disorders as well as identifying specific targets for intervention. While we focus on these categories, the CASPER model can be readily applied to any psychiatric disorder associated with social dysfunction (see Table 1).

We posit that the CASPER model can be used to disentangle the specific processes that make up cognition-based neurophenotypes of disorders by providing a model for testing each step in social processing. Specifically, one can envision a battery of short social cognitive tasks that can assess what functions are intact within the model, or what networks are being used to carry out specific functions. This battery can be coupled with naturalistic measurements to identify which processes are altered during actual social interactions, linking the highly controlled measures often collected in research to real social behaviors. Finally, this information can be contextualized in the child’s specific early experiences and social context. A better mapping of what specific cognitive processes have gone awry in individuals or in relation to specific symptoms or behavioral presentations of these disorders could help inform treatment approaches. Here, we use depression and conduct disorder as an exemplar to review what evidence there is for depression subtypes of functioning within each step of the CASPER model as well as proposed avenues for future research to test these hypotheses.

## CASPER and internalizing disorders

Prior research suggests that anxiety, mood, and trauma disorders are largely characterized by alterations in cue detection and attention to relevant cues, as well as differences in affect/motivation and experience-based social concept development. Specifically, internalizing disorders are also each associated with poorer emotion labeling skills [156–158] and negative interpretation and attentional biases [95, 120, 122, 159–161]. Furthermore, there is evidence for differences in SAL network size in individuals with depression [162] as well as alterations in DMN, VAN, and SAL function in individuals with depression [163], anxiety [96, 164], bipolar [165], and PTSD [166]. These disorders are highly heterogenous, co-occurring, and overlapping in symptomology [153, 167–169], however, making it difficult to identify syndrome-level differences in social cognition or determine how much overlapping social dysfunction is attributable to symptom overlap. Indeed, an analysis of symptoms in the DSM-5 found that of the top 15 most non-specific symptoms (i.e., symptoms that repeated the most across chapters), all but 3 were depression symptoms with only suicidality repeating fewer than 5 times [169]. Depression researchers are therefore increasingly seeking to characterize psychobiological models of depressive symptoms, capturing subgroupings of individuals with similar cognitive neurophenotypes [170, 171].

### Depression cognitive neurophenotyping

**Fig. 3:**
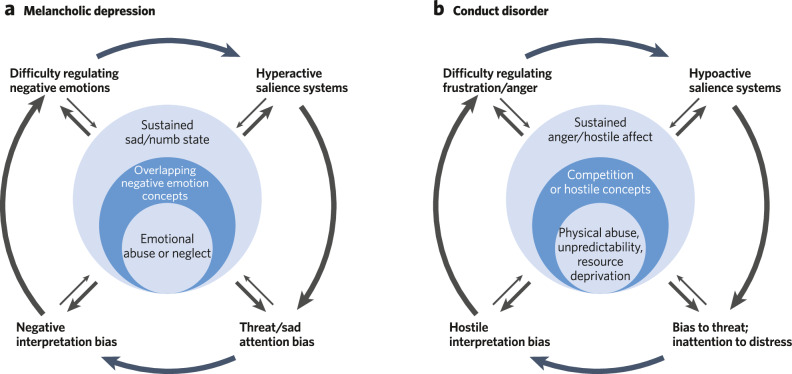
Theoretical models of how the CASPER model can be used to model social dysfunctions. **a** In this exemplar of a testable hypothesis for melancholic depression, the model links early emotional abuse or neglect to observed downstream emotion processing biases (i.e., attentional and interpretational) and alterations in emotion regulation. **b** In this exemplar of a testable hypothesis for conduct disorder, the model links resource deprivation and abuse to alterations in downstream attentional processing of others negative emotions and difficulty regulating anger.

While neurocognitive and psychosocial models of depression have been previously proposed [172, 173], there remains significant limitations surrounding their ability to explain the vast heterogeneity present in depressive phenotypes. Depression is a syndrome defined as presenting with at least one of two cardinal symptoms (depressed mood; anhedonia)—or three for youth (depressed mood; anhedonia; irritability)—and at least five symptoms out of a total of nine for at least two weeks. These criteria mean that depressed individuals can present with any of 256 symptom combinations, and two people with depression could share only one symptom [66]. In youth, not only is there an additional cardinal symptom that adds heterogeneity (irritability), but the frequency of symptoms presented in adolescents vs. adults differs notably, with adolescents endorsing more physical symptoms and adults endorsing more cognitive symptoms [174]. Co-occurrence with other disorders also differs with age of onset of depression. For example, children with preschool-onset depression are more likely to also present with ADHD as compared to adults with an adolescent or adult age of onset, while rates of co-occurring anxiety disorders are comparable across age cohorts [175–177]. Coupling this heterogeneity in presentation with the unique genetic and/or environmental etiology of each affected person’s depressive symptoms [168, 178, 179], a cognitive systems level approach that can meaningfully parse depression heterogeneity is critically needed. There are diverse social phenotypes that have been reported in depressed individuals that are at times contradictory. For instance, consider a situation where a depressed teen wants to stay out late, and their caregiver does not allow them to. The teen may respond to the caregiver’s limitations by shutting down and withdrawing, engaging in self-destructive behaviors, with frustration or defiance (irritability), or with excessive reassurance seeking for the perceived offense of asking. None of these are adaptive social responses to the blocked goal, and each appear to be a result of distinct cognitive processes. The CASPER model can be used to isolate *which* specific process may be characteristic of specific symptom clusters, helping to differentiate commonly co-occurring disorders and inform treatment approaches (Fig. 3a). Further, it’s an open question whether characterizing the upstream processes is enough to predict downstream behaviors, if neurocognitive profiles generalize, or if each person has their own unique profiles. The goal of CASPER is to characterize the full neurocognitive developmental profile to better identify patterns that explain symptoms.

Prior literature has supported the notion that depression arises from a combination of genetic and environmental factors, specifically adverse life experiences [41, 180], with a heritability liability of approximately 37% [178]. Given the protracted development of systems related to different components of social processing, when and how individuals face adversity likely has a strong influence on which CASPER process is most affected. For instance, adversity experienced before the age of six years has a disproportionate influence on hippocampal development [181]. Literature has found that the hippocampus has the fastest rate of development in infancy and early childhood [182–184], suggesting that influencing hippocampal development may be one way that early adversity can influence downstream social cognitive development conferring risk. Indeed, smaller hippocampal volumes have been observed across more than a dozen depressed cohorts with an even larger reduction in volume in individuals with an earlier depression age of onset [185]. Further, the preschool period is an important period for developing foundational social skills including emotion reasoning and mentalizing which coincides with maturation of the DMN [45, 186–188]. Taken together, this suggests that one mechanism conferring heterogeneity in depression phenotypes is individual differences in the long-term encoding of emotion concepts learned during the early childhood period.

There is some evidence for alterations in emotion concepts in individuals with depressive symptoms from the emotion differentiation and face labeling literature. Specifically, individuals with greater symptoms are more likely to have poorer differentiation of their own emotions (i.e., reporting feeling similarly across varied negative emotion inductions or contexts) and worse performance in labeling others’ emotions [189, 190]. While this area of research is relatively new, these recent findings suggest many possible avenues to explore to better understand this phenomenon. First, it is likely that the specific kind of negative early experiences could influence *why* there is poorer emotion discrimination later. For example, childhood emotional abuse (e.g., name calling) and neglect (e.g., not addressing emotional needs) are predictive of later depression more so than physical abuse or neglect [41], suggesting that the negative social cues are more closely linked to depression emergence than stress broadly. Further, unpredictable caregiving environments in infancy are associated with greater symptoms later in childhood [191, 192], suggesting that a cascade linking relative difficulty in predicting sensory and social signals can influence later emotional functioning. Taken together, it suggests that some children who develop depression-related social dysfunctions may have experienced fundamentally altered statistically learning in youth linking emotion cues to cause and consequence, resulting in misinterpretations of others’ ambiguous cues. Children experiencing emotional abuse likely learn quickly to interpret ambiguous cues as potential threats, or to link negative emotions that are typically interpreted distinctly (such as sadness and anger) to overlapping causes and consequences, which we predict is linked to DMN (and eventually widespread) alterations. Children who experience unpredictable environments, alternatively, may also have poorer concept discrimination for a different reason, such as alterations in sensation influencing salience processing. Longitudinal studies characterizing how early emotion-specific statistical learning, early caregiving experiences, and neurodevelopment are needed to test this development cascade in the CASPER model.

Prior work has demonstrated mixed directionality in studies of attentional biases in depressed individuals, which may be explained by the high co-occurrence of anxiety and depression. The CASPER model offers some possible testable explanations for this. Individuals with an anxious depression, for example, may have alterations in the cue detection phase of CASPER. Both prenatal factors and unpredictable postnatal sensory signals from caregivers may alter how cue salience is shaped across development. Prior literature has shown that maternal depression—a strong predictor of offspring depression [193]—is associated with offspring attentional and interpretational biases as well as hyperactivity of the amygdala during emotion processing [194–197]. It is possible that maternal emotion dysregulation during pregnancy can alter the development of the cue detection systems—which have a high concentration of HPA axis related receptors [198, 199]—and in turn hyperarousal of SAL and VAN in the infant.

## CASPER and externalizing disorders

Externalizing disorders in both youth and adult populations are characterized by disruptive behaviors, impulse-control difficulties, and impairments in social affiliation. The syndromes themselves are diverse, and certain disorders may be more reflective of deviations in only one domain (ADHD, for example, is primarily linked with impulse-control problems) whereas others may show deviations across two (e.g. Intermittent Explosive Disorder is linked with disruptive behaviors and impulse control problems) or all domains (e.g. substance use, oppositional defiant disorder, conduct disorder, and aggressive personality syndromes such as Antisocial and Borderline Personality Disorder). Similarly to internalizing disorders, alterations in cue detection and attention to relevant cues are consistently reported for externalizing syndromes, as well as differences in affect/motivation and experience-based social concept development profiles. Importantly, some of these symptoms may stem from cognitive processes not inherently social in nature (i.e. FPN and impulsivity) but may still impact social functioning. Many of these syndromes have common symptomology [200], suggesting the CASPER model may be integral in identifying cognitive neurophenotypes for each and identify unique (or shared) intervention points. As such, we highlight how the CASPER model may be applied to Conduct Disorder below.

### Conduct disorder cognitive neurophenotyping

Conduct Disorder is characterized by persistent antisocial behaviors, including violence and violations of moral norms [201]. Conduct disorder affects ~3% of the population [202], poses a significant clinical burden [203], and is associated with higher rates of attention, mood, and anxiety disorders [204]. Symptom expressions vary, but can include interpersonally exploitive tendencies, callous-unemotional behaviors and responses, impulsivity, and importantly, violent and damaging acts [205]. Conduct disorder youth often come to the attention of clinicians because of persistent peer aggression, both in terms of targeted, proactive aggression (e.g. bullying) and reactive, uncontrolled aggression (e.g. frustration, fighting), often presenting severe emotional deficits (e.g. remorselessness, callousness). Prior research into conduct disorder and related conditions has focused on deviations in empathic processing and deviations in attention networks [206, 207] but are not exclusive to these domains. In fact, application of the CASPER model may provide novel intervention points to consider in the etiology, diagnosis, and treatment for conduct disorder and related diagnoses (e.g., Oppositional Defiant Disorder) and phenotypes (e.g. Borderline features, bullying, psychopathy).

While youth with conduct disorder show deviations across CASPER processes, much of the literature has focused on underlying affect processing, which likely lead to downstream cue detection (e.g., distress in others) limitations. In turn, youth with conduct disorder may then also show limited attending to, and shallower social processing of, social information in others. Conduct disorder diagnosis strongly predicts adult psychiatric impairment [208], with 50% of youth with conduct disorder developing antisocial personality disorder [209]. These behaviors have detrimental effects on development for both those diagnosed with conduct disorder [210, 211] and those victimized [212]. The financial cost of an individual with persistent conduct disorder is estimated to be millions of dollars [213]. Neurodevelopmental differences in onset age are particularly important in childhood-onset conduct disorder [214], with early and severe symptom presentation associated with persistent, antisocial behavior across the lifespan [215, 216].

Similar to depression, early adverse life experiences likely play an outsized role in the development of misconduct. For example, in preschool aged children, it has been shown that living in a resource deprived neighborhood is linked with increases in both bullying behaviors and in other resource-driven (e.g., stealing) misconduct, even when accounting for other symptoms and parent behaviors [217]. Other longitudinal studies point to the impact of unpredictable, erratic parenting behaviors and physical abuse on the expression of conduct symptoms [218]. This suggests that (a) exposure to adversity may lead those with underlying diathesis to engage in select behavioral expressions consistent with conduct disorder; and (b) environmental events are likely influencing the schemas that promote aggression long before these children begin expressing aggression. The fact that the behavioral phenotype may already be presenting as early as preschool suggests that our neurodevelopmental examinations need to start at much younger ages, with a mind towards identifying changes in brain function which may underlie these developments. For example, recent work examining neonatal functional connectivity patterns has identified that alterations between CON and regions typically implicated in DMN processing (e.g. medial prefrontal cortex) predicts externalizing symptoms and callousness through age 3 [219]. This suggests that prenatal and genetic factors may confer risk for children to develop altered social processing consistent with conduct disorder behaviors.

Linking alterations in the timecourse of cognitive network development to specific cognitive schemas and concepts will be challenging, but studies of aggression in older children, adolescents, and adults has already provided significant clues to how exposure to adversity impacts these cognitive frames. For example, exposure to uncertainty and threat may cause individuals to view their world as unpredictable, which may in turn promote behavioral adaptations to ensure the individual can acquire resources. In short, exposure to these types of adversity may lead to the development of a risky life-strategy [220], with individuals predisposed to aggression utilizing these antisocial behaviors to obtain or maintain resources in a world that they perceive as unsafe, unpredictable, and unproviding [221]. Significant work supports this hypothesis in adults across domains (work, romantic, social), with a broad finding of reduced affiliation [112] and an expectation of hostility, and willingness to act to protect oneself from others hostility, in social interactions [222, 223]. In youth, aggression can serve as a warning to other aggressive individuals [222] to steer clear of confrontation, and narrative studies with externalizing youth observe a willingness to harm others with the assumption that others will likely harm them if they do not act first [224]. We speculate that early forms of social aggression in conduct disordered youth may be reflective of conceptualizing the world as aggressive, with motivational and affective systems developing in step. Preliminary evidence shows that resource deprivation affects neural structure in children, with further work needed to establish specific changes that may underlie these affective developments [225].

Conduct disorder youth have profound deviations in affective processing, with some arguing that the diagnosis should largely be understood as an emotion disorder [226–231]. Children and adolescents with conduct disorder and related traits have shown hypoactivation in subcortical neural regions (e.g., the amygdala) to affective stimuli, particularly negative affective states, like distress and fear [207, 232–234]. There also appears to be deviations in regions and circuits typically associated with reward processing [235–240]. These aberrations in associative and/or reinforcement learning and both positive/negative affective processing is not always consistently observed. For example, affective deficits may be mitigated by task characteristics and enhanced motivation, while reward processing deviations may not cleanly reflect hypo- or hyperactivation to rewards but rather deviations in prediction errors. Some work suggests a tendency to prefer immediate rewards, while some suggest an increased desire for relative rewards – that is, rewards coming at the expense of peers – both of which is consistent with adult diagnoses [74, 241]. In summation, affective learning in conduct disorder could reflect limitations in an ability to process others’ emotions and correctly assess rewards (e.g., reduced salience of others’ emotions, altered motivation to parse others’ emotions) or could reflect adaptations where immediate rewards are prioritized and affective information which could impair the acquisition of such rewards are not prioritized for further processing.

The role of attention—both stimulus-driven and goal-directed—has been well-characterized in conduct-disordered youth. Conduct disorder youth show hyporesponsivity via neural and physiological responses to novel cues, suggesting a broad insensitivity impacting bottom-up attentional detection. For example, contingency changes need to be exaggerated for aggressive youth to recognize [242] and conduct disorder youth often have reduced “attentional blinks” to distracting cues [101]. Furthermore, conduct disorder youth show reductions in visual processing and somatomotor networks, potentially suggesting limited salience processing writ large [243], though this needs to be formally tested. Related physiological work in adult populations notes reduced arousal responses (e.g. startle responses [244]) along with decreased activity in SAL network areas and decreased engagement of SAL networks in the presence of other task demands [245, 246]. Deficits have also been observed in top-down focused attention processing, with conduct disorder youth showing relative hypoactivation in both DAN [247] and FPN [248] regions. Meta-analyses in adult samples similarly shows deficient top-down detection and hypoactivation in the FPN [246]. Interestingly, several behavioral studies run counter these findings, suggesting certain traits associated with conduct disorder may actually aid in identifying subtle cues of vulnerability in others and directing focus to said information [249–253]. Rather than an inability to read emotions in others, it may be that aggressive individuals usually are not motivated to focus on the distress cues of others; however, when it aligns with their goals, they may be just as adept as typically developing populations. This suggests that motivation plays an outsized and, we argue, understudied role in interpreting activation differences and attentional anomalies previously observed in aggressive populations. For example, several studies have either changed timing constraints or provided task directions [113, 254] which nullified previously observed deviations. We posit the CASPER model may be uniquely qualified to explore attentional deviations in specific contexts rather than as unitary constructs, which may be key to identifying specifically when dysfunction will be observed, and when “dysfunction” may reflect ability limitations rather than limited effort.

As previously discussed, it is likely that individuals with conduct disorder and related psychopathology may already be prone to view social interactions from a competitive standpoint and assume hostility from others. Unsurprisingly then, studies examining how such individuals interpret social information often finds aberrant behavioral and neural responses. It has been found that conduct disorder youth have reduced functional connectivity within the DMN [255] and between the DMN and affective regions [256]. While structural connectivity may be intact, consistent deviations are found with reduced DMN functional connectivity [243]. Similar findings in psychopathy suggest interpretation of moral information may be shallowly processed [257], consistent with theories of aggressive phenotypes being less focused on the states of others or in desiring affiliation. Decision-making studies of youth with conduct disorder have largely addressed two aspects of their social decision-making processes, reactive and proactive aggression. Reactive aggression is linked with impulsivity, whereas proactive aggression is goal-directed and requires planning and creating opportunities (or at the very least seizing on opportunities) to aggress. Work has shown that some individuals may use aggression as a coping mechanism to rejection, responding to perceived slights or insults with vengeful acts [258]. Furthermore, aggression may be chosen as a pathway to achieve goals [259], feel competent and powerful, and ensure one’s place in the social hierarchy [71]. For example, researchers have experimentally shown that individuals show improved mood when allowed the opportunity to aggress against others [244]. Other studies have found that when individuals are experimentally aggressed upon by a conspecific, that greater evoked activity in reward system regions corresponds to the intensity of the counter-attack. Furthermore, real-world histories of aggression are related to activity in reward regions while aggressing against others in experimental paradigms [75, 258, 260, 261], suggesting that some instances violence might be understood as a reinforced mechanism for goal achievement [262] in certain cases rather than resulting from a breakdown in a regulatory system [263]. This may or may not differ from other externalizing syndromes, where proactive aggression is less expected but where reactive aggression may be serving immediate regulatory/coping mechanistic purposes. The CASPER model could be integral to clarifying aggressive acts across diagnoses resulting from impulsivity, frustration, or reinforced goal-attainment.

## Future research directions

To test these theories, future research can be designed with the CASPER model in mind to both interpret current and retrospective symptoms, while also predicting future social cognitive neurophenotypic problems. Indeed, we hope that future researchers test each component of the model as well as the directionality of the effects. For example, in depression research, studies of infants of depressed caregivers can measure early social and sensory experiences to characterize which variations confer altered emotion learning and subsequent risk, while studies of older children can measure multiple cognitive processes, ensuring that each process is measured both with and without social context to parse whether individual core functions are intact. It is critical to conduct these studies with full psychosocial context, measuring neighborhood and family level variables of the child’s needs and supports. These approaches can help future clinicians and researchers determine if particular social alterations are due to a down or upstream process. For example, studies could differentiate goal-directed attentional processing during a naturalistic movie versus a classic attention task devoid of social context. If symptoms correlate to attentional performance on the task similarly to attention to character emotions in the movie, that suggests alterations in attentional processing itself may be conferring social dysfunction rather than a broader attentional deviation. This can be experimentally tested by implementing an attention-based intervention. If this results in downstream improvements in social functioning, then it suggests that alterations in attention were the principal contributor. Alternatively, if there are different associations between contexts such as no association in the classic task but an association in the movie condition, this suggests that the alteration may be in the affect and motivation process or cue detection phase, shaping the goal-directed attention phase. This can be experimentally tested by implementing interventions focused on changing affective state or cognitive goals in the moment and then observing downstream effects. Further, neuroimaging during naturalistic social conditions will provide unprecedented insight to the cognitive processes individuals are engaging during social cognition and can be used in conjunction with the behavioral measures mentioned above to characterize social cognitive neurophenotypes in depression. It is critical that, as a field, we translate cognitive neuroscience research tools such that clinicians can test these cognitive processes bedside and use neuroscience-integrated results to inform care.

Researchers can also use the CASPER model to better understand any specific behavior, not just broad phenotypes. For example, avoiding social situations is associated internalizing disorders and with multiple possible underlying motivation (e.g., desire to be alone versus desire to avoid high sensory experiences), prior experiences (e.g., low reward during social interactions versus getting overwhelmed in an unexpected crowd), and/or attentional processing tendencies during social interactions (e.g., negative interpretation bias versus lack of attention to the right cues for adaptive functioning). Like in Fig. 3, researchers can use the CASPER model to flesh out and test theories relating to a specific behavior. In the case of social avoidance, researchers can assay multiple underlying differences in cue detection, attention, interpretation, affect/motivation, concepts, and experiences that might underly individual differences in their specific measure of social avoidance. This way, a profile that considers the full context can provide a much richer insight to the phenomenon (e.g., is social avoidance of peers during school associated with differences in cue detection? Interpretation?). Once the model is filled, then it can be tested through intervention on the correlated steps (e.g., attention training for alterations in the cue detection phase or CBT for alterations in the interpretation phase).

In a typical presentation of a youth with externalizing symptomology, it is often true that interventions are not broached till aggression is already occurring. The CASPER model suggests that by the time individuals are responding to their cues with aggression, several upstream processes have already veered off the road. We propose that front-line professionals (e.g., pediatricians) and social resources (e.g. community psychoeducation programs, neighborhood rejuvenation projects) may need to take larger roles in assessing adverse events or risk factors impacting affective development, before they are impacting attentional and interpretation networks. Wellness visits for youth may want to increase focus on parent behaviors, neighborhood characteristics, as well as parent schemas (which may be transmitted to their child). Concurrently, researchers need to extend functional network research into younger populations, to provide both normative trajectories of affective development, as well as study timing impacts on downstream processes associated with adversity. Of note, this approach will likely be key for explaining deviations between conduct disorder and related externalizing phenotypes. For example, while those with borderline personality features are also prone to interpersonal aggression, their social motivations differ from those with conduct disorder, as they are characterized by a strong desire for social acceptance, although this desire is persistently perceived by the individual to be unmet and unattainable [264]. This suggests that while these two disorders are related [265], the CASPER model approach may illuminate where etiological divergences occur. Disruptive behaviors are often difficult to accurately identify, as children have limited awareness and parents may not have insight into why their children may be acting antisocially with peers, at school, or with siblings. Lab-based tasks have been increasingly utilized to measure children’s social and cognitive functioning, demonstrating the ability to assess frustration tolerance and impulse control issues (which likely stem from deviations in effortful control networks), as well as reduced prosocial and increased antisocial preferences (which may more directly be linked to social processing networks). While academic researchers can focus on establishing the reliability of said measures, clinicians, pediatricians, and schools could consider socio-cognitive assessments in the same ways that testing is already implemented to better identify higher-risk children. Furthermore, this type of research will likely provide even greater clarity to links between behavior and functional brain network activity and connectivity. While certain functional networks are implicated in specific cognitive processes, their function is diverse and diffuse; understanding how the maturation of these networks links back to behavior will provide further insight into the implicated cognitive components.

We have provided two exemplar diagnoses, descriptively highlighting how past findings fit within the differential processes of the CASPER model. It is important to note that we do not primarily view the added utility of our proposed model for describing past work but in providing avenues for the generation new hypotheses and testing/identify novel mechanisms underlying maladaptive behavioral phenomena. The etiology of psychopathology has typically been viewed through a variant of harmful dysfunction lens; we propose an agnostic approach to psychopathology, with the possibility that the behaviors we observe are reasonable short-term adaptations impacting specific neurocognitive developmental processes. In the case of conduct disordered youth, should we interpret the limited attention to others’ emotions as dysfunction or the end result of an adoption of a fast-life strategy? There are understandable reasons to view the behaviors as dysfunctional and aberrant, but if these behaviors serve evolutionarily understandable functions that align with observable neural developmental processes (i.e., the maturation of the default mode), our interventions may need to be reconsidered away from “fixing a broken process in the person” towards addressing the conditions that promoted this phenotype. An alternative approach to “treatment” could therefore be to consider the current social context underlying the condition. Such a paradigm shift may require us to re-imagine treatment targets away from the individual and be more focused at broader factors (e.g., neighborhood, school, or family/caregiver resources) or suggest both individual level targets need be complemented with a greater emphasis on the environmental inputs which are reinforcing observed neural and behavioral adaptations.

Importantly, we expect that the neural systems underlying different processes may be particularly malleable at different epochs of development. Therefore, tracing a particular behavioral phenotype back through the CASPER model will provide greater insight into where social processing may have veered off its normative path and identify opportunities to intervene on both primarily impacted, as well as secondary or compensatory, cognitive targets. For instance, we may be able to improve our understanding of *why* an individual may have difficulty detecting affective stimuli if we can link it to adverse experiences at particular developmental windows. Not only will this improve primary prevention efforts (e.g., ensuring processes develop normatively in unaffected individuals) but could also allow us to consider concurrent neurodevelopment, as well as downstream results. Recent efforts have been made to longitudinally study younger and younger populations—such as the Adolescent Brain and Cognitive Development (ABCD) study [266], The HEALthy Brain and Child Development (HBCD) study [267], and Human Connectome Projects (HCP) [268] which include child, neonate, and in-utero samples—which may allow for potentially greater understanding of developing neural processing in the future. It is important to note, however, that the representativeness of the large sample is key to identifying whose developmental trajectory is being included in these models and therefore for whom they can provide an interpretable comparison group for. We suggest these large-scale efforts be coupled with hypothesis-driven models, to best test how the behavioral problems we observe in our clinics can be best understood and empirically treated. Additionally, while we formulated CASPER as a cognitive network model, we will likely need multiple methodologies and levels of bran measurements (e.g., genetics, local population coding, multivariate models, etc.) to fully test CASPER.

Here, we present a testable model for explaining how social and emotional aberrations manifest, rooted in developmental cognitive neuroscience. The CASPER model links social information processing theories and research across cognitive neuroscience, social psychology, and developmental science disciplines to provide a testable model of how real-world social processing unfolds and is altered in individuals with psychopathology. We hope that this unified theory will spurn new research that informs future personalized interventions by targeting the upstream processes that are resulting in dysfunction. Finally, the developmental lens this model provides we hope will inform research into *when* to intervene, leveraging developmental periods when these systems are most plastic or targeting processes that are most malleable across development.
